# Corrosion Tendency of S235 Steel in 3.5% NaCl Solution and Drinking Water During Six Months of Exposure

**DOI:** 10.3390/ma17235979

**Published:** 2024-12-06

**Authors:** Daniela Laura Buruiană, Alina Crina Mureşan, Nicoleta Bogatu, Viorica Ghisman, Elena Emanuela Herbei, Vasile Başliu

**Affiliations:** 1 Interdisciplinary Research Centre in the Field of Eco-Nano Technology and Advanced Materials (CC-ITI), Engineering Faculty, Dunarea de Jos University of Galati, 47 Domnească Street, 800201 Galati, Romania; daniela.buruiana@ugal.ro (D.L.B.); nicoleta.simionescu@ugal.ro (N.B.); viorica.ghisman@ugal.ro (V.G.); elena.herbei@ugal.ro (E.E.H.); 2 Cross-Border Faculty, Dunarea de Jos University of Galati, 47 Domnească Street, 800201 Galati, Romania; vasile.basliu@ugal.ro

**Keywords:** S235JR steel, surface morphology, corrosion behaviour, corrosion mechanism, roughness, Vickers hardness

## Abstract

The pipeline transport industry is constantly developing due to the high efficiency, long life, varied diameters of the pipelines, but a significant problem is the corrosion that occurs because of the corrosive attack of the various environments in which the pipelines are used. This study deals with the ex situ characterizations (optical microscopy, scanning electron microscopy coupled with energy dispersive X-ray analyses, X-ray diffraction analysis, roughness, and Vickers hardness analyses) and the in situ characterizations (gravimetric and electrochemical methods). Samples of steel were tested at immersion time, after 336 h, 672 h, 1344 h, 2016 h, and 4032 h of exposure to a 3.5% NaCl solution and drinking water. The corrosion rate evaluated with the gravimetric method varied between 0.036518 and 0.008993 mm/year in the 3.5% NaCl solution and 0.02834 and 0.034162 mm/year in drinking water. The electrochemical method resulted in an estimated corrosion rate range of 0.097–0.681 mm/year for the 3.5% NaCl solution, and 0.028–0.0472 mm/year for drinking water. The passivation effect, lifetime, and operating limit of the S235JR steel in the tested corrosive environments were studied. The study can provide technical support to increase the service life of S235JR steel and to predict the suitable environment reduce corrosion costs.

## 1. Introduction

Corrosion is known as the electrochemical or chemical reaction that happens between a material and its environments, which leads to a deterioration of the material and its characteristics. Corrosion reduces the bearing capacity of structures, reduces the lifecycle of steel, and causes significant economical loss [1,2,3,4]. For example, significant economic losses are registered every year [5] and the losses caused by metal corrosion reach 3.4% (estimated to be USD 2.5 trillion) of the annual gross domestic products (GDP) in industrialized countries [6]. 

S235JR steel is a very common structural steel used in many applications, including construction (high rise buildings/skyscrapers, shopping malls, offices, factories, houses, road barriers, bridges, and train tracks), industrial pipes, pipelines that supply gas, oil, water, agricultural machinery and construction equipment, the marine industry (marine hulls), and pressure vessel manufacturing [7,8,9,10]. Structural steel offers several advantages, such as excellent mechanical resistance and lower cost compared to other materials [11,12,13]. However, a significant drawback is its susceptibility to corrosion, which restricts its range of applications [14,15,16]. 

Karlsdóttira et al. [17] report corrosion behaviour of S235JR steel under severe conditions in a gas separation tower, recording a higher corrosion rate (2.99 mm/year and 4.25 mm/year for 4 weeks and 12 weeks testing time) using the gravimetric technique. This indicates severe uniform corrosion of the carbon steel occurring in the geothermal environment in the tower. Faes et al. [18] report corrosion behaviour of S235JR steel, austenitic stainless steel (UNS S31603), and duplex stainless steel (UNS31803) in an artificial geothermal brine. Tests conducted at 80 °C and 130 °C showed that carbon steel displays a high uniform corrosion rate initially, but the formation of a protective layer leads to a decrease in the average corrosion rate over time. The authors concluded that none of the materials studied performed perfectly under harsh geothermal conditions.

Some authors evaluated the corrosion behaviour of S235JR steel in a 20% NaCl solution [19], in 5%, 15%, and 20% NaCl at 3 °C, and in a 3.5% NaCl solution for 5 weeks of immersion [20] using the gravimetric method. The research results indicate that the weight loss of S235JR steel immersed in a 20% NaCl solution at ambient temperature is directly proportional to the duration of corrosion. Corrosion of S235JR in 5%, 15%, and 20% NaCl at 3 °C depends on time of corrosion and on the temperature of the environment. Quan and Xie [21] investigated the corrosion characteristics of Q235 steel and 16Mn steel in a sulphur-containing alkaline solution by weight loss and electrochemical methods using XPS, SEM, and EDS techniques. The authors conclude that the weight loss method indicated a higher corrosion rate for Q235 steel compared to 16Mn steel. Electrochemical results reveal that the current density for Q235 steel is greater than that of 16Mn steel, and the corrosion kinetics of both steels are governed by a combination of charge transfer and ion diffusion processes.

In addition, for the corrosion of carbon steel, an important parameter that must be considered is the corrosion rate. Some experimental research and data reveal the corrosion behaviour of S235JR steel in an atmospheric environment [22,23,24]. The authors concluded that the corrosion rate follows a pattern of increase, decrease, and then increase, suggesting that the passivation film on the surface of Q235 low-carbon steel, along with the rupture of this film after the formation of the matrix rust layer, provides some corrosion resistance. Some researchers argue that under turbulent conditions, surface roughness and the friction coefficient on carbon steel surfaces increase, leading to a higher corrosion rate as corrosion products accumulate [25,26]. Although there is a wealth of experimental data and research on the corrosion rate of low-carbon steel in various environments under harsh conditions, low-carbon steel pipes must undergo pickling and passivation before being used in actual production. Many studies focus on the morphology and corrosion rate of S235JR steel over short periods under severe conditions or in high-temperature atmospheric environments.

In our study, we evaluate and compare the morphological aspects and corrosion behaviour of S235JR carbon steel in two different environments: a 3.5% NaCl solution and drinking water (from the drinking water supplier of Galati city—APA CANAL S.A. Galati, Romania) at different immersion times (at immersion time, after 336 h, 672 h, 1344 h, 2016 h, and 4032 h of exposure in corrosive mediums). The morphology of the steel surfaces before and after corrosion tests was evaluated using optical microscopy and scanning electron microscopy. To better understand the corrosion process, the corrosion products formed on the S235JR steel surfaces in the tested environments were examined using the EDX method. Additionally, to evaluate the corrosion products formed on the steel samples obtained after immersion, we used XRD analysis. The roughness and Vickers hardness of the samples were evaluated to observe the influence of immersion time in two tested corrosive environments on the mechanical properties of S235JR carbon steel. To evaluate corrosion rate, we used the gravimetric technique and electrochemical methods (open circuit potential, polarization resistance, corrosion rate). In order to estimate the changes in the physico-chemical parameters of the corrosive solutions on testing time, some properties of the corrosive solutions such as pH, potential, conductivity, total dissolved solids, and salinity were measured.

The novelty of the paper consists of studying the morphological aspects of the steel surfaces at different periods of immersion and the corrosion behaviour of S235JR steel in 3.5% NaCl and drinking water for a long period of time (using weight loss and electrochemical methods). Also, some physico-chemical parameters of the corrosive solutions during corrosion were evaluated to identify the influence of the corrosion products passing into the solution on these parameters.

## 2. Materials and Methods

Steel specimens were cut from a S235JR steel plate (purchased from Mairon Galati, Galati, Romania) with a chemical composition (wt.%) of 0.17 C, 0.028 P, 0.025 S, 0.025 Si, 0.12 N, 0.45 Cu, and 1.40 Mn, and balanced by Fe. The samples used for the weight loss testing measured 50 mm × 30 mm × 3 mm and the electrochemical measurements’ testing surface active area was 2.5 cm^2^ (the specimens were sealed in epoxy glue). The exposed area of steel used for corrosion testing was abraded with 600, 800, and 1200 grit silicon carbide papers, degreased with acetone, dried in air, and stored in a desiccator for 24 h before testing. 

### 2.1. Ex Situ Characterizations

An optical microscope type Kern OBE 114 (Kern and Sohn Optics, Balingen, Germany) and a scanning electron microscope (SEM) type TESCAN VEGA 3 LMH (Tescan, Brno, Czech Republic) was used to observe and analyze the surface morphology of a sample’s surface during each stage of the experiment (initial time, after 336 h, 672 h, 1344 h, 2016 h, and 4032 h of exposure to corrosive environments). The accelerating voltage of SEM was 20 kV. SEM was combined with an EDX elemental X-analyser to qualitatively analyze the chemical composition of the corrosion products formed on the tested surfaces and explore the growth process and the corrosion mechanism of the corrosion products on the surface of S235JR. Also, XRD measurements were performed. Every specimen was tested at least three times to ensure the reproducibility of the experiments. The XRD measurements were carried out using DRON 3M powder XRD diffractometer with a Co target, operating at 30 kV and 20 mA, a 2θ = 15°–90° measurements range, a step = 0.05°/s, and a total time per sample of 2 h and 13 min.

The Insize ISR-C002 roughness tester (Insize Co., Suzhou New District, Suzhou, China) was used to evaluate the surface roughness of the steel surfaces before and after the corrosion tests with high accuracy. For roughness, we used various international parameters. The following were considered the most important for this research: R_a_—the arithmetic average of the absolute values of the profile deviations from the mean line of the roughness profile, R_z_—mean roughness depth, R_q_—mean peak width, and R_t_—the total height of the roughness profile [27,28]. 

The measurement of Vickers hardness of the tested materials was conducted via the Vickers method (HV), using the Digital Micro-Vickers hardness ISR-C100 (Insize Co., Suzhou New District, China). An indenter was used in the form of a diamond pyramid, whose load varied from 0.02 to 20 N. The applied indentation loading was 0.5 kg at the top of the tested samples surfaces. The indentation depth was approximately 3 µm. The results for roughness and Vickers hardness were obtained as three values with an average of three measurements for each sample.

### 2.2. In Situ Characterizations

Corrosion weight loss measurements: The predominant technique for the practical applications, gravimetric method, or weight loss method involve measuring mass loss by immersing a material sample in a corrosive medium (immersion test). In essence, the gravimetric methods for measuring corrosion rates are simple and do not require the measurement of currents or voltages [29]. The S235JR specimens were placed in beakers filled with the corrosive environments: a 3.5% NaCl solution and drinking water (300 mL of each solution). All the experiments were conducted at 328K without aeration. Before the tests and after 336 h, 672 h, 1344 h, 2016 h, and 4032 h of exposure to the corrosive environments, the samples were weighted with Kern EWJ 300-3H analytical balance (a precision of 0.001 g). The weight of the specimens was evaluated with procedures outlined in ASTM G 31, “Standard Guide for Laboratory Immersion Corrosion Testing of Materials” [30] and Standard ISO 11845:2020 [31]. The exposed surface was cleaned with absolute ethanol and then dried using a stream of high-purity nitrogen gas. Any remaining organic solvent was removed in a vacuum oven. Three parallel experiments were conducted for each sample to determine the average weight loss [32,33]. The corrosion rate was evaluated conforming to ASTM G1-90 (1999) [34] and using Equation (1).

(1) $$CR(mm\;/year)=\frac{\Delta W\cdot k}{A\cdot t\cdot d}$$

where CR means the corrosion rate, mm/year; K is a constant depending on the unit of measurement of the corrosion rate (8.76 × 10^4^); ΔW is the mass loss (difference between initial weight of samples before corrosion tests and sample weight after different exposure time in various environments), g; A is the surface area of the material tested for corrosion in cm^2^; t is the exposure time of the material to corrosion; and h and d represent the density of the corroding material in g/cm^3^ (7.874 g/cm^3^ for iron). 

Electrochemical measurements: Open circuit potential (OCP), polarization resistance (R_p_), and corrosion rate (CR) were used to evaluate the corrosion behaviour of the tested samples using a Voltamaster 4—PGP 201 connected to a PC with the VoltaMaster version 5.10 software. Electrochemical measurements were made at immersion time and after immersion time (336 h, 672 h, 1344 h, 2016 h, and 4032 h) in 150 mL of the corrosive solutions. The tests were conducted in a three-electrode cell, with the working electrode composed of S235JR steel. The geometric surface area of the working electrode was 2.5 cm^2^, and the rest of the electrode surface was sealed off from the electrolyte using epoxy glue. Prior to each measurement, the electrode surface was cleaned with distilled water and dried at room temperature. It was then immersed in the test solution to begin the measurement. A saturated calomel electrode (SCE = +241 mV/NHE) served as the reference electrode, while the counter electrode, made of 99.8% platinum (surface area 0.5 cm^2^), was used. The supporting electrolytes (the corrosive environments) were obtained by dissolving NaCl p.a. reagents (from Sigma-Aldrich, St. Louis, MI, USA, 99% purity) to obtain a 3.5% NaCl solution, and drinking water was used from the drinking water supplier of Galati city—APA CANAL S.A. Galati. To evaluate the polarization resistance and the corrosion speed at different initial immersion time periods, OCP was performed for each sample for 60 min at an average period of 0.6 s; 40 points were determined, scan rate = 1 mV/s, over voltage = 40 mV, and the OCP duration between each R_p_ and V_corr_ for the 40 points was 1 min. The electrolyte was not deoxygenated. 

The corrosion rate of a metal in a corrosive environment is generally evaluated by the gravimetric method [35], but it requires a long exposure time in a corrosive environment and does not allow the instantaneous evaluation of the corrosion speed. On the other hand, electrochemical methods are fast and accurate and thus are of great importance for practical applications. Linear polarization requires short measurement times, has unlimited sensitivity, and allows for continuous corrosion monitoring. The disadvantage is that it disturbs the system by imposing an external bias, which can cause changes in the specific properties of the system [36].

The reproducibility of the conducted investigations is based on an average of three samples for each specimen type. The physico-chemical parameters of the corrosive environments are provided in Table 1.

### 2.3. Using Physico-Chemical Parameters to Monitor Corrosive Solution During Corrosion Tests

To better understand the corrosion process and to establish the chemical reactions between the tested material and the corrosive solutions, we analyzed the physico-chemical parameters of the testing corrosive environment before and after the corrosion process (336 h, 672 h, 1344 h, 2016 h, and 4032 h). Parameters (pH, potential, conductivity, total dissolved salts, and salinity,) were measured using a multi parameter analyser Phoenix Instrument, multiEC-15 (Phoenix Instrument GmbH, Heinkelstr. 430827 Garbsen, Deutschland). 

## 3. Results and Discussion

### 3.1. Morphological Aspects 

**Figure 1:**
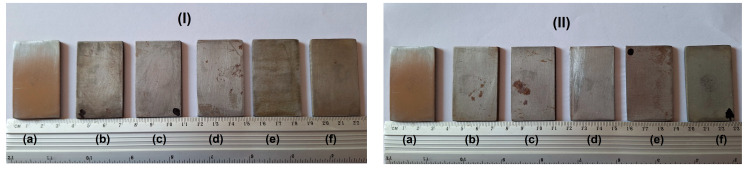
Visual aspects of tested samples in 3.5% NaCl (**I**) and drinking water (**II**) before and after corrosion tests: initial (**a**), after 336 h (**b**), 672 h (**c**), 1344 h (**d**), 2016 h (**e**) and 4032 h (**f**).

The colour and grain size of rust can offer insights into the corrosion rate of steel. The progression of rust is categorized into levels, specifically for rusts that are over nine years old (this classification does not apply to rusts of a younger age). The visual aspects of the tested samples before and after different immersion times in corrosive environments are presented in Figure 1. It can be observed that the colour of the samples is different depending on the tested solution and the immersion time; the S235JR steel samples corroded in 3.5% NaCl more than in drinking water.

**Figure 2:**
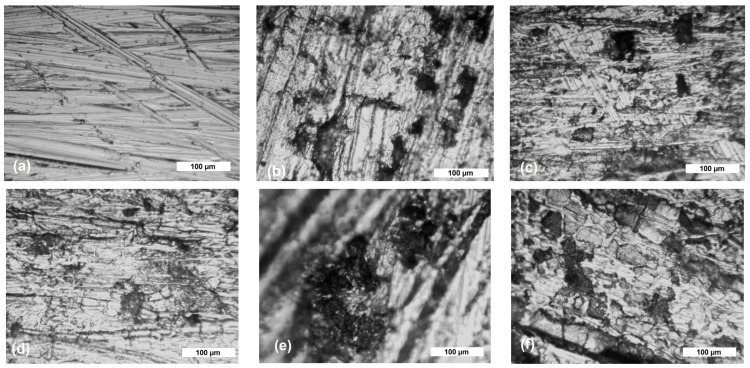
Optical micrograph of S235JR steel before (**a**) and after 336 h (**b**), 672 h (**c**), 1344 h (**d**), 2016 h (**e**), and 4032 h (**f**) of immersion in 3.5% NaCl solution (at magnification ×50).

**Figure 3:**
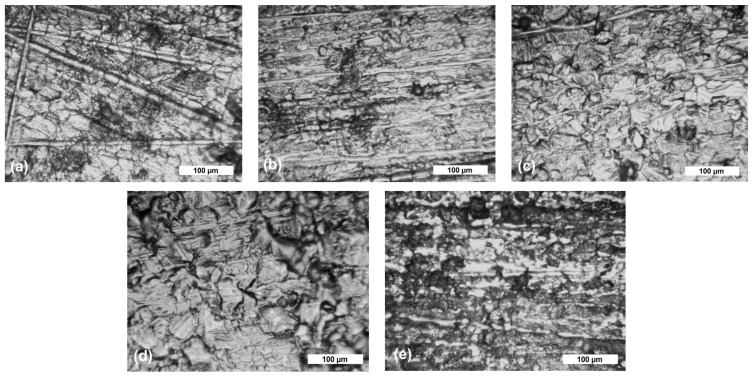
Optical micrograph of S235JR steel after 336 h (**a**), 672 h (**b**), 1344 h (**c**), 2016 h (**d**), and 4032 h (**e**) of immersion in drinking water (at magnification ×50).

To highlight the corrosive attack, Figure 2a–f and Figure 3a–e show the optical micrographs of the steel samples’ surfaces (at the top of the tested surfaces) before and after different exposure periods in corrosive solutions. From the obtained micrographs, it can be observed that before immersing the samples in the 3.5% NaCl solution and drinking water, the surfaces are clean, without traces of corrosion products or defects.

An analysis of the images after immersing the samples in the 3.5% NaCl solution and drinking water reveals the presence of corrosion products (rust) formed on the samples’ surfaces. The S235JR steel sample immersed in the 3.5% NaCl solution shows the most significant corrosion, with visible micro- and macro-pitting caused by the electrolytic action of Cl^−^ ions. Both testing solutions induced localized corrosion—pitting corrosion (a deep wide pit indicating that the iron suffers pitting corrosion), which was more predominant in the 3.5% NaCl than in drinking water and general corrosion. The visible pits on the S235JR steel are a result of an autocatalytic process. Localized dissolution of steel is one of the most common and destructive causes of structural failure, with the surface oxides of S235JR mainly composed of iron (III) oxides. When the steel’s passive protective film is compromised, oxidation of the underlying iron occurs, leading to increased acidity within the pits. This process is driven by a half-cell reaction, where the pits function as the anode and the metal surface as the cathode. Consequently, Cl^−^ ions diffuse into the pits to preserve charge neutrality, balancing the excess positive charges produced by the oxidation of the metal [37,38].

Two types of morphologies of corrosion products can be observed. The first one is the annular corrosion observed more predominantly in drinking water, where each inclusion was surrounded by the annular corrosion product. More corrosion sites were observed with increasing corrosion time, and the size of the “circles” increased as well. The other type of morphology is cystiform, which were made up of the catenarian corrosion products [39].

**Figure 4:**
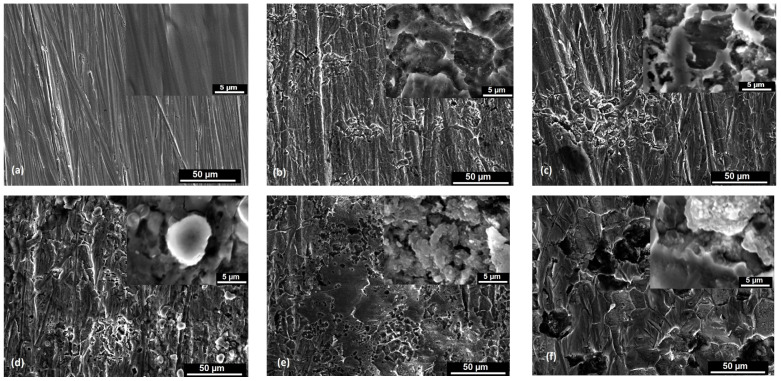
SEM images of S235JR steel samples before (**a**) and after 336 h (**b**), 672 h (**c**), 1344 h (**d**), 2016 h (**e**), and 4032 h (**f**) of immersion in 3.5% NaCl. Scale bar: 50 μm. Inset shows higher magnification images at scale of 5 μm.

**Figure 5:**
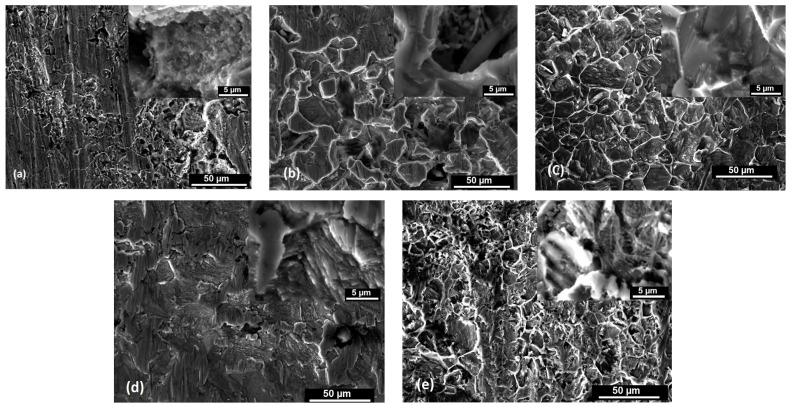
SEM images of S235JR steel samples after 336 h (**a**), 672 h (**b**), 1344 h (**c**), 2016 h (**d**), and 4032 h (**e**) of immersion in drinking water. Scale bar: 50 μm. Inset shows higher magnification images at scale of 5 μm.

The scanning electron microscopy images of the S235JR steel plates (at the top of the tested surfaces) before and after exposure to the 3.5% NaCl solution and drinking water for 336 h, 672 h, 1344 h, 2016 h, and 4032 h are shown in Figure 4a–f and Figure 5a–e. According to the corrosion degree and depth, the corrosion products on the surface of the S235JR steel were dispersed and distributed randomly.

By examining the microscopic morphology of S235JR steel using a scanning electron microscope, we observed that all corrosion products induce cracks on the surface. These cracks allow ions to reach the steel surface, continuing the corrosion process [40,41]. As shown in Figure 4a, the steel surface prior to corrosion exhibits some abrasive scratches. In the initial stages of corrosion, the corrosion products reduce the corrosion rate (Figure 4b and Figure 5a,b), but corrosion continues to progress (Figure 4c,e and Figure 5d,e). The corrosion products form in layers, suggesting that multiple layers have developed on the surface of the specimen due to ongoing corrosion. The microscopic images reveal that solid corrosion products are deeply embedded within the surface of the next layer, with the surrounding area appearing darker, indicating the formation of a dense, protective corrosion product layer. After 336 h of immersion in the tested solutions, it can be observed that pitting corrosion appears and the surfaces have cracks and pits. After 672 h of immersion, the steel surface is more corroded and many loose and porous corrosion products were observed. In the 3.5% NaCl solution, it can be observed that the surfaces are more corroded than in the drinking water, pits are deeper, and a lot of accumulation of corrosion products formed on the steel surfaces. After 1344 h, the surface is filled by corrosion products that act like a passive film, but after 2016 h and 4032 h of immersion in the tested solutions, the surface of S235JR is more corroded and has general and pitting corrosion. Parts of the corrosion products detached from the steel surface and went into the solution, as shown on the images obtained with the electron scanning microscope where they appear as black areas due to the gaps left by the corrosion products. These aggregates grow and rupture to form new corrosion products. 

The microscopic images in Figure 4c,e,f and Figure 5c–e reveal a significant accumulation of corrosion products on the steel surfaces. Variations in the thickness of the corrosion products suggest differing corrosion reaction rates at the corrosion interface. The corrosion products displayed distinct porous, fractured, and delaminated characteristics with uneven surface corrosion. The prominent corrosion pits and cracks indicated localized corrosion. Throughout the experiment, the specimens exhibited a dense and uniform corrosion product layer on their surfaces. The corrosion products formed a thin, even layer that loosely covered the carbon steel surface.

### 3.2. Analysis of Corrosion Products 

**Figure 6:**
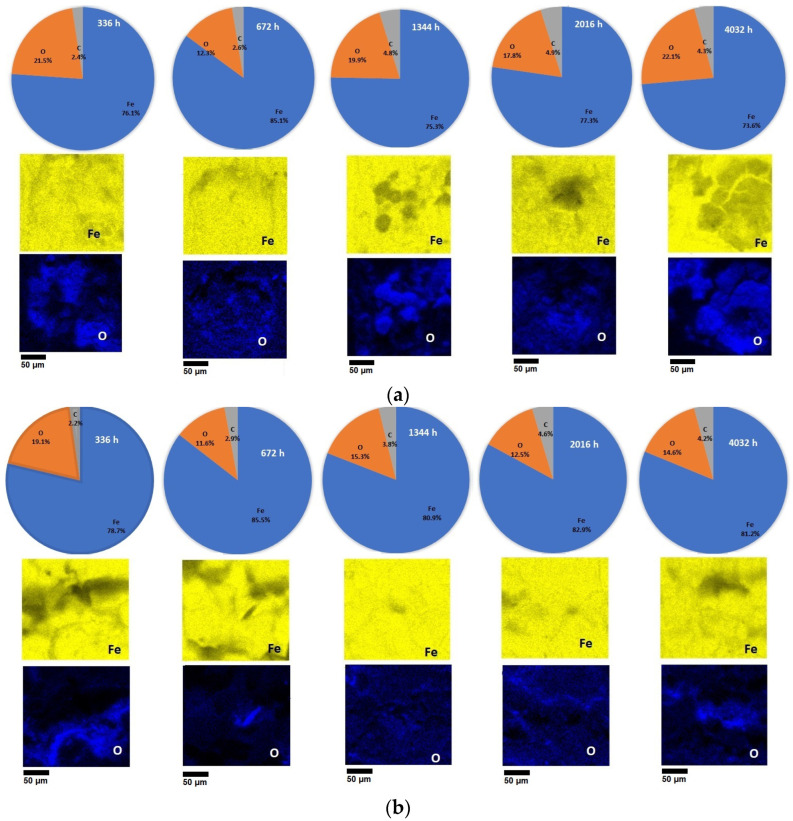
Content of Fe, O, C, and surface mapping obtained from EDX spectra of S235JR steel surfaces after different time of immersion in 3.5% NaCl (**a**) and drinking water (**b**).

The composition of the steel surface was investigated by energy-dispersive X-ray (EDX) spectroscopy before and after corrosion in the 3.5% NaCl solution and drinking water for a period of 336 h, 672 h, 1344 h, 2016 h, and 4032 h of immersion. The results obtained are presented in Figure 6. In an EDX spectrum, each element produces a distinct set of peaks, and the peak intensities can be used to calculate the elemental composition ratio in the sample.

The results indicate that the elemental composition of the corrosion products was affected by the different test environments. Generally, rust consists of various types of oxides, including hydrated oxides, oxyhydroxides, and other crystalline and amorphous substances, with the primary components being iron and oxide [42]. The main elements in the corrosion products were iron and oxygen, indicating that the main components of the corrosion products under the different test conditions were iron matrix, oxyhydroxides, and iron oxides. Similar results were also reported by Sundjono [43], Royani [44], and Gedge [45]. A higher oxygen content is observed for the samples tested in the 3.5% NaCl solution (the values of the oxygen content were between 12.3 wt.% and 22.1 wt.%) compared to the samples tested in the drinking water (the values of the oxygen content were between 11.6 wt.% and 19.1 wt.%) because the NaCl solution is a much more corrosive agent and the attack on the surfaces of the samples is more intense, causing the formation of corrosion products in higher concentrations. 

According to the results presented in Figure 6, it can be observed that in the 3.5% NaCl solution and in the drinking water, there is the same trend regarding the variation in the oxygen content during the corrosion tests. The oxygen content decreased sharply from 336 h to 672 h of immersion (a variation from 21.5 wt.% to 12.3 wt.% in the 3.5% NaCl solution, and from 19.1 wt.% to 11.6 wt.% in the drinking water, respectively). Then, there is an increase at the 1344 h (the oxygen content in the samples tested in NaCl reached 19.9 wt.% and reached 15.30 wt.% in the drinking water to), after which the oxygen content decreases at 2016 h in the samples tested in both corrosive solutions (in the 3.5% NaCl solution, the oxygen content reached 17.8 wt.% and reached 12.5 wt.% in drinking water). Following that, at the end of the corrosion tests after the 4032 h of immersion, the oxygen content for the steel samples tested in the 3.5% NaCl solution was 22.1 wt.% and for the steel samples tested in the drinking water, it was 14.6 wt.%. The sudden decrease in the oxygen content for the S235JR steel samples was observed in the first part of the corrosion tests because the oxide layer initially formed on the surfaces, which ensures the protective role against corrosive agents becomes unstable, and the soluble compounds formed pass into the solution and can no longer protect the steel surface. 

During the corrosion tests from 1344 h to 4032 h, the variation in the oxygen content was not very large, but the fluctuation was maintained because the corrosion products form on the surface, then crumble and pass into the solution, form again on the surface until they can also have a protection and passivation role, and the phenomenon of corrosion appears much more accelerated. If the rust layer is thinner and layered, it is difficult to effectively prevent the corrosion process, even though the rust layer is dense. However, in the immersion environments tested, after a certain immersion time interval (336 h in the 3.5% NaCl solution and 672 h in the drinking water), the rust layer was thicker, which slowed down the corrosion process, resulting in the low corrosion rate.

While the impact of extended immersion time on the elemental composition of the corrosion products is relatively minimal, the combination of the EDX and SEM results reveals that the composition of the corrosion products did change, a finding that is also confirmed by the XRD method.

**Figure 7:**
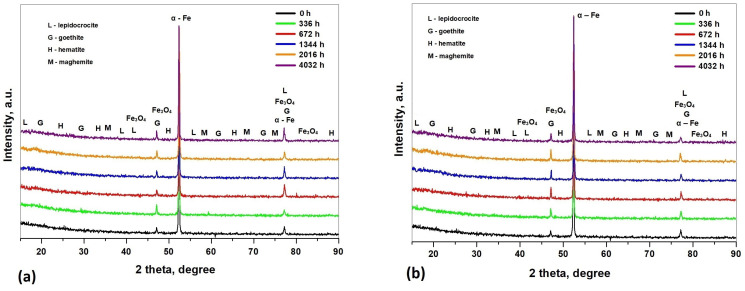
XRD pattern of S235JR steel surfaces before and after corrosion tests in 3.5% NaCl (**a**) and in drinking water (**b**).

As can be seen from the XRD data presented in Figure 7, the main component is α- Fe and the corrosion products that predominate are represented by α-FeOOH (goethite), γ-FeOOH (lepidocrocite), ${Fe}_{2}O_{3}$ (maghemite and hematite), and ${Fe}_{3}O_{4}$. Lepidocrocite facilitates the dissolution of the dissolved solvents because this material has a feathery structure. From the XRD spectra, it can be observed that the immersion time and type of the corrosive medium do not influence the presence of different corrosion products; these parameters influence the composition of the corrosion products. Furthermore, the corrosion products of the S235JR steel in the 3.5% NaCl solution and drinking water were similar.

In the initial stage of corrosion, the corrosion products formed on the steel surface are thin and sporadic, but the amount of the corrosion products increases with the increasing immersion time in the corrosive environment. Due to the increasing immersion time, lepidocrocite was transformed into goethite and ${Fe}_{3}O_{4}$, whereas lepidocrocite was unstable and was converted to goethite.

As is known, the corrosion process is mainly controlled by the cathode process in the sodium chloride solution because in this type of environment the permeability and mobility of oxygen is low. Under the action of the NaCl solution, the cathodic and anodic reactions occurring in the active zone in the initial immersion experiment corrosion test are iron dissolution (Equation (2)) and oxygen reduction (Equation (3)) [46,47].

(2) $$Fe\to {Fe}^{2+}+{2e}^{-}$$

(3) $$O_{2}+H_{2}O+{4e}^{-}\to 4{HO}^{-}$$

${Fe(OH)}_{2}$ is oxidized into ${Fe(OH)}_{3}$ in the drinking water environment. The increase in corrosion rate determined the formation of ${Fe}_{3}O_{4}$ by the depletion of oxygen. Lepidocrocite formed rapidly at the surface of the specimens in the solution with pH between 6.0 and 8.0. ${Fe}^{2+}$ is suspended on the surface of lepidocrocite, promoting further dissolution of lepidocrocite and conversion to goethite and ${Fe}_{3}O_{4}$ with a decrease in the pH value. The main reaction processes are presented in Equation (4) as follows:

(4) $$2Fe+O_{2}+2H_{2}O\to {2Fe(OH)}^{+}+{2HO}^{-}$$

Since the ${Fe(OH)}^{+}$ ions were unstable, they could react with oxygen to generate an Fe(OOH) film. This was followed by a dehydration reaction, γ-Fe(OOH) → γ-${Fe}_{2}O_{3}$, resulting in the formation of a stable iron oxide [48]. These substances were identified using XRD spectroscopy. The excess positive charge and acidity were increased by the ${Fe}^{2+}$ hydrolysis, which occurred in anodic areas at the steel/rust interface through Equations (5)–(7).

(5) $${4Fe(OH)}^{+}+{4HO}^{-}+O_{2}\to 4\beta -FeOOH+{2H}_{2}O$$

or

(6) $${4Fe(OH)}^{+}+{4HO}^{-}+O_{2}\to 4\gamma -FeOOH+{2H}_{2}O$$

(7) $$2{Fe}^{2+}+\frac{1}{2}O_{2}+3H_{2}O\to 2\gamma -FeOOH+4H^{+}$$

γ-FeOOH would also be reduced to ${Fe}_{3}O_{4}$ by ${Fe}^{2+}$, and ${Fe}_{3}O_{4}$ can also be directly formed from metals in an oxygen-deficient environment through the following reactions (Equations (8)–(10)) [49,50]:

(8) $$2\gamma -FeOOH+{Fe}^{2+}\to {Fe}_{3}O_{4}+2H^{+}$$

(9) $$3Fe+4H_{2}O\to {Fe}_{3}O_{4}+4H_{2}$$

(10) $${Fe}_{3}O_{4}+O_{2}\;\to \;\gamma -FeOOH$$

The goethite formed is thermodynamically stable in structure and can work as a protective layer toward steel corrosion (Equation (11)).

(11) $$\gamma -FeOOH\overset{H^{+}or\;O_{2}}{\to }\alpha -FeOOH$$

Goethite forms a dense protective layer to steel corrosion that improves corrosion resistance because lepidocrocite is assumed to consist of smaller particles than goethite and akageneite (*β*-FeOOH). The stable goethite phase, obtained from the reduction in lepidocrocite, decreases with time, and the corrosion rate is expected to increase again [51,52].

The cathodic sites are confined to the outer layers, restricting the diffusion of oxygen to the underlying metal surface as the rust layers thicken. A significant change in the anodic reaction rate is observed after the 1344 h for the samples immersed in the testing solutions, indicating a general decrease in the corrosion rate. This decrease is likely due to the formation of the less soluble goethite rust phase.

### 3.3. Mechanical Properties 

Deterioration of the mechanical properties of steel is an important consequence of corrosion. A change in steel behaviour can lead to unexpected problems and even to undesired brittle failure due to a reduced cross section. So is important to study corrosion impact on the mechanical properties of S235JR steel in the 3.5% NaCl solution and drinking water in order to make a correct assessment of the corroded steel. Table 2 presents the values of different parameters of steel surface roughness (R_a_, R_z_, R_q_, R_t_) before and after corrosion tests during 336 h, 672 h, 1344 h, 2016 h, and 4032 h in the tested solutions. 

As can be seen from the values presented in Table 1, the lowest value of roughness was obtained for the sample of the S235JR steel before immersion (average roughness with a value of 0.833 ± 0.003 µm). Overall, the R_a_ values are highest for samples corroded in 3.5% NaCl than in drinking water because sodium chloride is a more corrosive medium than water, and the corrosion products formed on the steel samples will determine a greater roughness on the surfaces. Factoring in immersion time, the average roughness increases in both tested solutions with a greater value for the samples tested in the 3.5% NaCl solution (1.869 ± 0.016 µm in 3.5% NaCl and 1.110 ± 0.009 µm in drinking water) after 336 h of immersion. After 672 h and 1344 h, the values of R_a_ decreases in both tested solutions until a value of 1.121 ± 0.015 µm in 3.5% NaCl and 0.985 ± 0.004 µm in drinking water are reached. After 2016 h of immersion, a slight decrease in roughness in 3.5% NaCl (0.982 ± 0.006 µm) and a slight increase for the sample tested in drinking water (1.002 ± 0.006 µm) is observed. Finally, after 4032 h of immersion, the value of the average roughness increases greatly in both tested solutions, reaching 1.442 ± 0.018 µm in the 3.5% NaCl solution and 1.303 ± 0.012 µm in the drinking water. 

From the presented data, it can be observed that for both tested solutions, the values of average roughness do not present a constant tendency to increase or decrease during time. The results show that the corrosion products formed on the surfaces can inhibit the corrosion of S235JR carbon steel in the tested solutions to some extent, but the corrosion of the samples surfaces still exists. Increases in the roughness can be attributed to an increase in the corrosion processes because the roughness influences the corrosion potential, favouring the appearance of corrosion pits [53]. The corrosion products deposited on the steel surfaces show that lower roughness is associated with surfaces where the corrosion products are evenly distributed across the entire steel surface, forming flat, compact, and protective layers. In contrast, higher roughness values correspond to surfaces where the corrosion products form loosely compacted layers, resulting in an uneven distribution. The surfaces of the S235JR steel with high roughness exhibit protrusions, likely caused by the release of corrosion products or the formation of porous corrosion layers due to the interaction of corrosion solutions with the surface. These protrusions can create pathways or channels that facilitate the transportation of corrosive species to the substrate thereby increasing the corrosion rate.

The Vickers hardness before and after the corrosion tests (336 h, 672 h, 1344 h, 2016 h, and 4032 h) in 3.5% NaCl and drinking water are presented in Table 3.

The Vickers hardness of the tested samples in the 3.5% NaCl solution are in the range of 119.0 ± 1.3 HV0.5 and 102.9 ± 0.9 HV0.5 and for the S235JR steel samples tested in drinking water, the Vickers hardness is in the range of 136.2 ± 01.2 HV0.5 and 102.1 ± 1.1 HV0.5. It can be observed that compared to the Vickers hardness of the samples before testing (149.5 ± 1.5 HV0.5), the values of the hardness decrease by about thirty units for the samples tested in 3.5% NaCl and by about ten units for the samples tested in the drinking water after 336 h of immersion in the corrosive environments. Between 336 h and 4032 h of immersion in 3.5% NaCl solution, the Vickers hardness was maintained an almost constant value (a difference of about fifteen units) and in the drinking water, the difference in the Vickers hardness was about thirty units. It can be determined that the small fluctuations appear because the distribution and size of the corrosion products that form on the S235JR steel surfaces are not sufficiently uniform. 

### 3.4. Analysis of Corrosion Weight Loss

**Figure 8:**
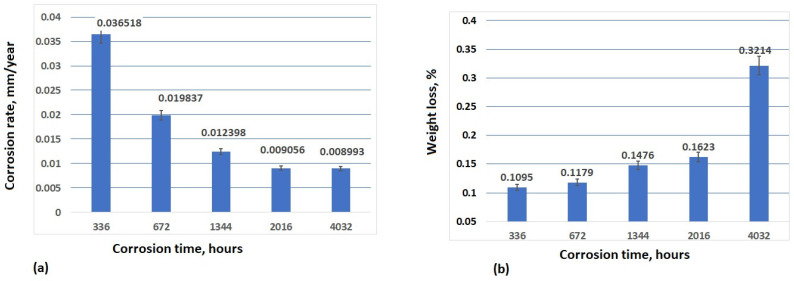
Corrosion rate (**a**) and weight loss (**b**) of S235JR tested samples after different time of immersion in 3.5% NaCl solution. Results obtained using gravimetric method.

**Figure 9:**
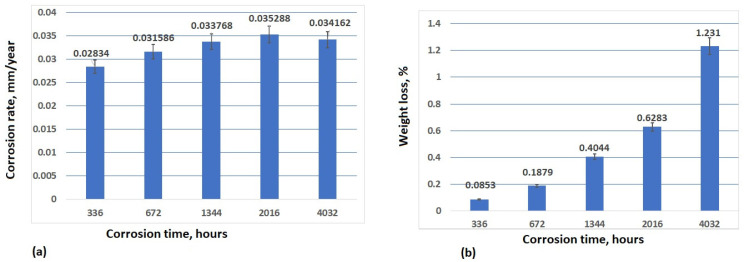
Corrosion rate (**a**) and weight loss (**b**) of S235JR tested samples after different time of immersion in drinking water. Results obtained using gravimetric method.

Figure 8 and Figure 9 show the corrosion rate, as well as the weight loss of the samples tested in 3.5% NaCl and drinking water, respectively.

The immersion tests in the corrosive environments were carried out in order to study the corrosion behaviour of S235JR steel over a long period of time. As can be seen, after 336 h of immersion in 3.5% NaCl, the corrosion rate is 0.036518 mm/year, but after 672 h of immersion in 3.5% NaCl, the corrosion rate sharply decreased until 0.019837 mm/year, and this downward trend continued for up to 1344 h and 2016 h of immersion (0.012398 mm/year and 0.009056 mm/year, respectively). After 4032 h of immersion, the corrosion rate was almost constant with a lower difference of 0.00063 mm/year. The weight loss increases with the increasing corrosion time. In 3.5% NaCl, the weight loss increases slowly in time until 2016 h (0.1095% after 336 h, 0.1179% after 672 h, 0.1476% after 1344 h, and 0.1623% after 2016 h of immersion in 3.5% NaCl). The main reason for this pattern may be the presence of corrosion products on the specimens’ surfaces, which can decrease the corrosion rate during longer periods of immersion [54]. After 4032 h of immersion, a higher value of weight loss (0.3214%) can be observed because the passive film of the oxide protective layer was destroyed, and corrosion was accelerated. Two diffusion processes are observed: diffusion through the corrosion layer and mass transport in the liquid phase. The extent to which these processes are coupled depends on the immersion time. Oxygen transport occurs not only in the liquid phase but also through the porous layer of the corrosion products. An increase in the weight loss rate can be attributed to the influence of chloride ions on the dense passive film. According to [55,56], these ions penetrate the passive film and disrupt its integrity only when a sufficiently high concentration gradient is established on the surface, which takes some time.

**Figure 10:**
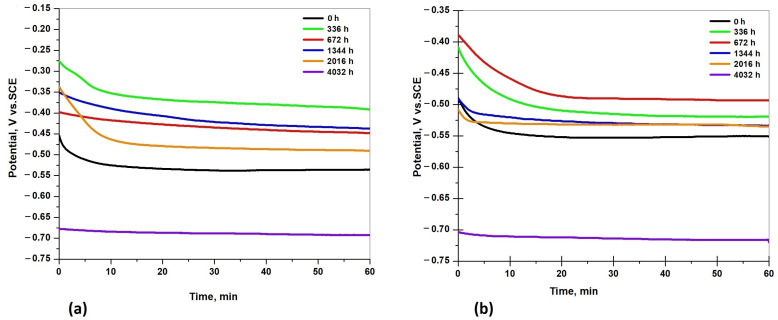
Open circuit potential of S235JR steel samples in 3.5% NaCl (**a**) and drinking water (**b**) before and after corrosion tests.

The corrosion rate in the drinking water was a value between 0.002834 mm/year after 336 h of immersion and 0.034162 mm/year after 4032 h of immersion. As can be seen from Figure 10a, the corrosion rate increases slowly after 672 h (0.031586 mm/year), 1344 h (0.033768 mm/year), and 2016 h (0.035288 mm/year). The slight increase in the corrosion rate in the drinking water indicates that the passivation film constituted by the corrosion products forms a better protective layer on the substrate compared to the first corrosion-resistant layer during the early stage of corrosion. This phenomenon is associated with the electrochemical protection mechanism of the passive film. It is primarily linked to the gradual thickening of the rust layer and the increased proportion of stable α-FeOOH components. As the second corrosion-resistant layer formed after the passivation film, it progressively reduced the exposure of the substrate to corrosive media and decreased the contact area, leading to an increase in resistance to electrochemical reactions. After 4032 h of immersion in the drinking water, the corrosion rate slightly decreases, reaching a value of 0.034162 mm/year. 

Corrosion of iron (the main component of S235JR steel) is initiated by pitting and spreads as layers of corrosion products form, but not all layers are adherent to the surface of the iron, as some of the layers have less adhesion because they are porous and the role of the occluded pits is negligible as compared to the porous film effect [57]. 

### 3.5. Electrochemical Measurements

#### 3.5.1. Open Circuit Potential 

Figure 10a,b presents the open circuit potential (OCP) values of the S235JR steel immersed in testing solutions (3.5% NaCl and drinking water) before and after corrosion tests for a period of 336 h, 672 h, 1344 h, 2016 h, and 4032 h of immersion.

The OCP (corrosion potential) is a qualitative parameter and gives information about the thermodynamic stability of the sample surface in a particular corrosive environment. This potential varies with time due to changes in the oxidation tendency of the surface. In both testing solutions, the OCP values increased to more negative values in the first few minutes of immersion, but stabilized after 25–30 min. After one hour of immersion in the 3.5% NaCl solution and drinking water, the OCP values of all the tested samples were stable. The highest negative value of OCP was recorded for the samples immersed 4032 h in the corrosive solutions: −0.69 V in the 3.5% NaCl solution and −0.71 V in the drinking water (which indicate that after 4032 h of immersion, the tested samples are thermodynamically more susceptible to corrosion). 

For the steel samples immersed in 3.5% NaCl, the tendency of corrosion potential is as follows: OCP increased after 2016 h, 672 h, and 1344 h, and the highest value of OCP was recorded for the samples immersed 336 h in the 3.5% NaCl solution (−0.392 V). The OCP values are more negative until 336 h because the compact layer of the corrosion products still exists on the surface and acts like a protective film, but after a long period of immersion, the corrosion products were removed from the surfaces and become more susceptible to corrosion (the more negative values for OCP were recorded for the samples immersed between 336 h and 4032 h).

The tendency of the open circuit potential in the drinking water is as follows: the OCP increased after 2016 h and was approximatively equal with the OCP after 1344 h of immersion, which increased after 336 h, and the smallest value of OCP was recorded for the samples immersed 672 h in the drinking water (−0.493 V). The shift to fewer negative values of the open circuit potential in both testing solutions indicates that the corrosion products formed on the steel surfaces reduced the corrosion susceptibility until 4032 h of immersion when the products could no longer protect the surfaces. The tendency of the OCP is to increase or decrease with immersion time without a linear trend, as corrosion products form and then pass into the solution, where pitting and localized corrosion induce fluctuations in the OCP values. It is generally understood that a lower OCP indicates greater electrochemical activity and more intense corrosion in the case of carbon steel [58].

#### 3.5.2. Polarization Resistance

**Figure 11:**
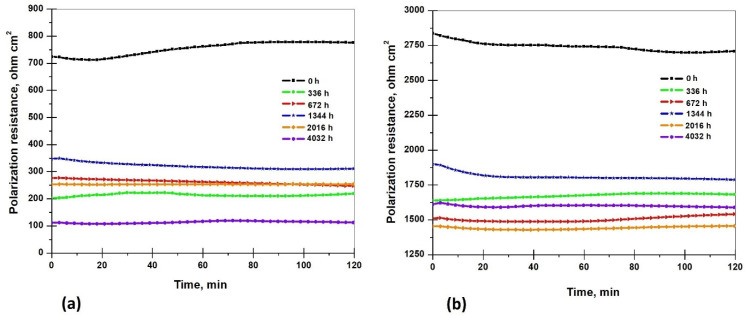
Polarization resistance of S235JR steel samples in 3.5% NaCl (**a**) and drinking water (**b**) before and after corrosion tests.

The Rp method is a real-time, direct corrosion monitoring technique that allows for the measurement of the corrosion rate. The corrosion current calculated using this method represents the current that flows at the metal–electrolyte interface when the metal is immersed in the corrosive medium [59]. Figure 11a,b presents the polarization resistance values of the S235JR steel immersed in the 3.5% NaCl solution and drinking water before and after the corrosion tests for a period of 336 h, 672 h, 1344 h, 2016 h, and 4032 h of immersion.

From Figure 11, it can be observed that polarization resistance is higher in the drinking water than in the 3.5% NaCl solution, which means the corrosion rate will be less in the drinking water than in the 3.5% NaCl solution. For samples tested in 3.5% NaCl, it can be observed that R_p_ is smaller before immersion compared to R_p_ in the drinking water by about four times, with a value of 776.78 Ώ·cm^2^, and 2709 Ώ·cm^2^, respectively. After different times of immersion in the 3.5% NaCl solution, it can be observed that the values of R_p_ have fluctuations so that after 336 h of immersion, the value of R_p_ was 218,984 Ώ·cm^2^, after 672 h it was 247,727 Ώ·cm^2^, after 1344 h it was 311.12 Ώ·cm^2^, after 2016 h it was 253,967 Ώ·cm^2^, and after 4032 h of immersion the value of R_p_ was 112.54 Ώ·cm^2^. During testing, the polarization resistance increased between 336 h and 1344 h with 28.743 Ώ·cm^2^ and 63.39 Ώ·cm^2^ because the corrosion products that form on steel surfaces act as a protective film at the first stage of immersion. For the S235JR steel samples tested in the drinking water, it can be observed that R_p_ is higher before immersion, with a value of 2709.8 Ώ·cm^2^. After different times of immersion in the drinking water, it can be observed that the values of R_p_ have fluctuations so that after 336 h of immersion, the value of R_p_ was 1681.9 Ώ·cm^2^, after 672 h it was 1541.0 Ώ·cm^2^, after 1344 h it was 1787.6 Ώ·cm^2^, after 2016 h it was 1456.0 Ώ·cm^2^, and after 4032 h of immersion the value of R_p_ was 1589.9 Ώ·cm^2^. 

During the corrosion tests in both testing solutions, it can be observed that the polarization resistance does not have a tendency to increase or decrease consistently over time. The values of Rp fluctuate because the corrosion system is a dynamic system, and corrosion products are formed on the steel surfaces, which passivate the surface, but after different periods of time, they enter the solution. Corrosion products are reformed on the surface of the steel until the protective films formed by hydroxides and oxides only have a protective role, and then localized corrosion and pitting appear, which influence the polarization resistance values over time.

#### 3.5.3. Corrosion Rate

**Figure 12:**
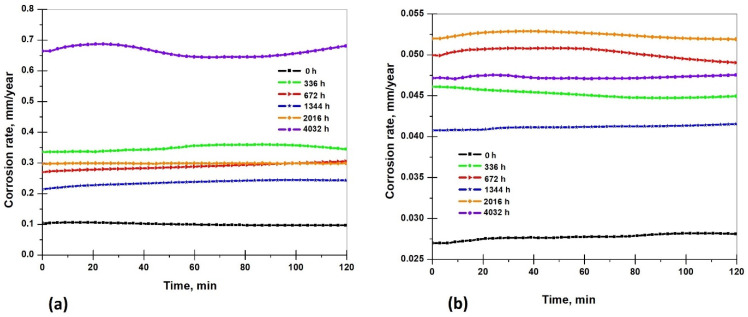
Corrosion rate of S235JR steel samples in 3.5% NaCl (**a**) and drinking water (**b**) before and after corrosion tests. Results obtained using electrochemical method.

The corrosion rate of the S235JR steel immersed in the 3.5% NaCl solution and drinking water before and after the corrosion tests for a period of 336 h, 672 h, 1344 h, 2016 h, and 4032 h of immersion is shown in Figure 12a,b.

The corrosion rate of the S235JR specimens in the 3.5.0% NaCl solution and drinking water shows a fluctuation with the increasing corrosion time, which reached a maximum of 0.681 mm/year at 4032 h in the 3.5% NaCl solution and 0.053 mm/year at 2016 h in the drinking water. In the 3.5% NaCl solution, the corrosion rate before immersion had a value of 0.098 mm/year, the first increase occurs in 336 h (0.344 mm/year), then it decreases in the 672 h (0.305 mm/year) and in the 1344 h (0.243 mm/year), and after that, in the 2016 h, it increases slowly (0.298 mm/year) and reaches the maximum value (0.681 mm/year) at 4032 h. The surface corrosion products formed gradually through accumulation; however, the presence of Cl^−^ ions easily triggered pitting corrosion, which led to a reduction in the protective corrosion product layer. As the amount of corrosion products decreased, they became increasingly involved in the corrosion process, driving further corrosion and ultimately increasing the corrosion rate [60].

In the drinking water, the final corrosion rate of the experiment shows the following pattern: before immersion (0.028 mm/year) < 1344 h (0.041 mm/year) < 336 h (0.045 mm/year) < 4032 h (0.047 mm/year) < 672 h (0.050 mm/year) < 2016 h (0.053 mm/year). A higher difference at the corrosion rate values can be observed at 1344 h (0.013 mm/year), but after that, the difference between corrosion rate values is slower (between 0.002 mm/year and 0.004 mm/year).

In an oxygen and water environment, iron (II) hydroxide was readily oxidized to iron (II) hydroxide. The high corrosion rate resulted in oxygen depletion and induced the formation of iron (II, III) oxide. 

The corrosion rates determined using the gravimetric method were notably lower than those obtained through the electrochemical methods, as the electrochemical techniques offer an immediate measurement of the corrosion rate. The use of the electrochemical methods, which involve disrupting the system with an external signal, can lead to higher observed corrosion rates [61].

### 3.6. Physico-Chemical Parameters of Corrosive Solutions During Corrosion Tests 

Some physico-chemical parameters (pH, potential, conductivity, total dissolved salts, and salinity) of the solutions after the corrosion process (336 h, 672 h, 1344 h, 2016 h, and 4032 h) are presented in Table 4 and Table 5.

For the 3.5% NaCl solution, the pH shows the following pattern: 0 h < 672 h < 336 h < 1344 h < 2016 h < 4032 h. From the initial value until 672 h of immersion, the pH has a lower acidity, after that, the pH became lower, basic. The potential of the 3.5% NaCl solution decreases after 336 h of immersion, but increases slowly after 672 h and then rapidly after 1344 h, 2016 h, and 4032 h of immersion of the testing samples. The conductivity increases slowly until 1344 h and then decreases slowly in time. The values of the total dissolved solids and salinity of the 3.5% NaCl solution with the corrosion products show the following pattern: 4032 h = 2016 h < 336 h < 672 h < 1344 h < 0 h.

For the drinking water with the corrosion products, the pH maintains a low basicity trend during tests and shows the following pattern: 336 h < 1344 h < 0 h < 2016 h < 672 h < 4032 h. The variation in pH of the drinking water with the corrosion products that dissolved from the steel surface and came into the solution is very low, with a variation of 0.64 ± 0.1 pH units between the lowest and the highest values during the test periods. The potential of the drinking water during the tests has a very slow variation with only 19.7 ± 0.1 mV. The conductivity decreases slowly until 672 h, then increases slowly, decreases at 1344 h, and then increases slowly again. The values of salinity of the drinking water with the corrosion products show the following pattern: 2016 h < 1344 h < 672 h < 4012 h < 336 h < 0 h.

The pH variation of the 3.5% NaCl solution with the corrosion products is determined by a higher corrosion rate of the steel samples in this testing solution. As the pH increases from 7 to 10, the weight loss is found to increase, as does the level of tuberculation. In contrast, the corrosion products decrease at higher pH values. Iron oxides are formed that remain on the steel surface and are insoluble in corrosive solution; the iron concentration decreased when the pH was raised from 9.40 to 10.06. The lower variation in the pH values of the drinking water with the corrosion products indicates a lower corrosion rate; the solution is more stable than the 3.5% NaCl solution. 

The values of the conductivity and total dissolved solids evaluated in 3.5% NaCl show an increase and then a decrease during the immersion time; the iron from specimens dissolved in the solution separates into charged particles (ions) that conduct electricity. In the drinking water, these physico-chemical parameters decrease slowly in time because the corrosion rates are slower than in the 3.5% NaCl solution and the iron dissolved slowly.

## 4. Conclusions

In this work, the morphological characterization and corrosion behaviour of the S235JR steel in the 3.5% NaCl solution and drinking water at immersion time, after 336 h, 672 h, 1344 h, 2016 h, and 4032 h of exposure to corrosive mediums were investigated and compared. The roughness and Vickers hardness were evaluated during the tests. The main conclusions from the experiments are as follows:

(1) From the morphology analysis, it can be concluded that the surfaces most affected by corrosion products was the samples immersed in 3.5% NaCl where visible micro- and macro-pits appear due to the electrolytic action of ${Cl}^{-}$ ions. Both testing solutions induced localized corrosion—pitting corrosion and general corrosion. The visible pits on S235JR resulted from an autocatalytic process.

(2) EDX analysis revealed that the primary elements in the corrosion products were iron and oxygen, suggesting that the main components of the corrosion products under the various test conditions were the iron matrix, oxyhydroxides, and iron oxides. These findings were further confirmed by XRD analysis, which identified α-Fe, α-FeOOH, γ-FeOOH, ${Fe}_{2}O_{3}$, and ${Fe}_{3}O_{4}$. For both corrosive environments, the same trend regarding the variation in the oxygen content during the corrosion tests exists. The immersion time and the type of the corrosive medium do not influence the presence of different corrosion products; these parameters influence the composition of the corrosion products. 

(3) The determined average roughness does not show a constant trend of increasing or decreasing over time. The obtained results show that the corrosion products formed on the surfaces of the tested steel can inhibit the corrosion to some extent, but the corrosion of the sample surface still exists. For the Vickers hardness, there are small fluctuations in the determined values because the distribution and size of the corrosion products that form on the S235JR steel surfaces are not uniform enough.

(4) The weight loss increases with the increasing corrosion time. In 3.5% NaCl, the weight loss increases slowly in time until 2016 h, but after 4032 h of immersion, a higher value of weight loss can be observed because the passive film of the oxide protective layer was destroyed, and corrosion is accelerated. The corrosion rate in the drinking water measured by the gravimetric method increases slowly during 20016 h of immersion, but after 4032 h, a slight decrease was observed.

(5) In both testing solutions, the open circuit potential values stabilize after 25–30 min of immersion. During the corrosion tests in both testing solutions, it can be observed that the polarization resistance and corrosion rate do not have a tendency to increase or decrease consistently over time. The values of the polarization resistance and corrosion rate fluctuate because the corrosion system is dynamic.

## Figures and Tables

**Table 1:** Physico-chemical parameters of corrosive environments.

Corrosive Environment	pH	Potential	Conductivity	Total Dissolved Solids	Salinity
3.5% NaCl	5.77 ± 0.02	73.82 ± 0.12 mV	57 ± 0.06 mS/cm	41.4 ± 0.07 ppt	38.1 ± 0.40 g/L
Drinking water	7.94 ± 0.03	−52.2 ± 0.08 mV	1057 ± 53 μS/cm	751 ± 15.30 ppm	0.523 ± 0.06 g/L

**Table 2:** Roughness parameters for tested S235JR steel samples.

Tested Solutions	Immersion Time, h	Roughness Parameters, µm			
		R_a_	R_z_	R_q_	R_t_
	0	0.833 ± 0.003	8.276 ± 0.213	1.098 ± 0.006	9.509 ± 0.231
3.5% NaCl	336	1.869 ± 0.016	13.692 ± 0.371	2.5360 ± 0.026	17.471 ± 0.744
	672	1.401 ± 0.014	10.443 ± 0.456	1.9190 ± 0.014	13.078 ± 0.658
	1344	1.121 ± 0.015	8.151 ± 0.195	1.439 ± 0.008	9.160 ± 0.314
	2016	0.982 ± 0.006	6.998 ± 0.054	1.252 ± 0.007	8.611 ± 0.206
	4032	1.442 ± 0.018	9.155 ± 0.202	1.816 ± 0.014	12.804 ± 0.473
Drinking water	336	1.110 ± 0.009	8.151 ± 0.081	1.424 ± 0.012	10.158 ± 0.362
	672	1.096 ± 0.005	8.820 ± 0.116	1.497 ± 0.013	11.531 ± 0.381
	1344	0.985 ± 0.004	7.662 ± 0.065	1.284 ± 0.026	10.832 ± 0.288
	2016	1.002 ± 0.006	6.908 ± 0.044	1.262 ± 0.025	8.860 ± 0.186
	4032	1.303 ± 0.012	8.750 ± 0.231	1.628 ± 0.038	10.058 ± 0.315

**Table 3:** Vickers hardness HV 0.5 for tested S235JR steel samples.

Vickers hardness, HV 0.5	Tested solutions	Immersion time, h					
		0	336	672	1344	2016	4032
	3.5% NaCl	149.5 ± 1.5	119.0 ± 1.3	112.4 ± 1.1	112.0 ± 0.9	109.0 ± 0.7	102.9 ± 0.9
	Drinking water		136.2 ± 1.2	127.9 ± 0.8	105.2 ± 0.9	104.0 ± 0.7	102.1 ± 1.1

**Table 4:** Physico-chemical parameters of 3.5% NaCl solution during corrosion tests.

Immersion Time	pH	Potential, mV	Conductivity, mS/cm	Total Dissolved Solids, ppt	Salinity, g/L
336 h	7.16 ± 0.5	−13.5 ± 0.1	57.5 ± 0.3	40.9 ± 0.2	37.3 ± 0.3
672 h	6.26 ± 0.2	38.6 ± 0.3	57.9 ± 0.2	41.0 ± 0.1	37.4 ± 0.3
1344 h	9.40 ± 0.3	438.2 ± 2.7	58.2 ± 0.4	41.3 ± 0.3	37.7 ± 0.2
2016 h	9.53 ± 0.3	448.5 ± 3.2	57.4 ± 0.2	40.7 ± 0.2	37.1 ± 0.1
4032 h	10.06 ± 0.4	481.2 ± 3.1	57.2 ± 0.1	40.7 ± 0.2	37.1 ± 0.1

**Table 5:** Physico-chemical parameters of drinking water during corrosion tests.

Immersion Time	pH	Potential, mV	Conductivity, µS/cm	Total Dissolved Solids, ppm	Salinity, mg/L
336 h	7.94 ± 0.6	−52.2 ± 1.6	1057 ± 10.6	751 ± 3.4	523 ± 2.3
672 h	7.77 ± 0.6	−44.0 ± 1.4	933 ± 8.6	663 ± 3.1	456 ± 1.9
1344 h	8.16 ± 0.5	−64.8 ± 1.6	832 ± 5.6	590 ± 2.3	405 ± 1.7
2016 h	7.86 ± 0.4	−50.9 ± 1.7	874 ± 6.2	527 ± 3.7	361 ± 1.3
4032 h	8.06 ± 0.6	−63.7 ± 1.8	704 ± 5.8	501 ± 2.9	342 ± 1.2

## Data Availability

Any data or material that support the findings of this study can be made available by the corresponding author upon request.
